# Experimental demonstration of a fully inseparable quantum state with nonlocalizable entanglement

**DOI:** 10.1038/srep45045

**Published:** 2017-03-27

**Authors:** M. Mičuda, D. Koutný, M. Miková, I. Straka, M. Ježek, L. Mišta

**Affiliations:** 1 Department of Optics, Palacký University, 17. listopadu 1192/12, 771 46 Olomouc, Czech Republic

## Abstract

Localizability of entanglement in fully inseparable states is a key ingredient of assisted quantum information protocols as well as measurement-based models of quantum computing. We investigate the existence of fully inseparable states with nonlocalizable entanglement, that is, with entanglement which cannot be localized between any pair of subsystems by any measurement on the remaining part of the system. It is shown, that the nonlocalizable entanglement occurs already in suitable mixtures of a three-qubit GHZ state and white noise. Further, we generalize this set of states to a two-parametric family of fully inseparable three-qubit states with nonlocalizable entanglement. Finally, we demonstrate experimentally the existence of nonlocalizable entanglement by preparing and characterizing one state from the family using correlated single photons and linear optical circuit.

Relations among quantum systems can be much more intimate than among everyday classical systems which we observe through our senses. If two states of two quantum systems with well defined and distinguishable local properties are superimposed, the individual properties are smeared out whereas the state as a whole still exhibits well defined global properties. The participating systems are then in a special relationship because although their local properties are uncertain, they are strongly correlated at same time, whence the respective states got a fitting nickname entangled states^1^.

Previous considerations are related only to pure states which represent an idealization of real states. For more realistic mixed states the entanglement is defined via a generalization of an equivalent characterization of pure-state entanglement, that is a property which cannot be created from pure product states using only local unitary operations. Thus, generalizing the notion of local unitaries to the set of local operations and classical communication (LOCC), which are more sensible operations in the context of mixed states, we can define a generally mixed entangled state as a state which cannot be created by LOCC^2^. Therefore, from the point of view of entanglement, the set of all states *ρ*_*AB*_ of two subsystems, denoted as *A* and *B*, divides into two disjoint subsets. One subset is given by entangled states whereas the other subset comprise the so called separable states, i.e., states which can be prepared by LOCC and therefore attain the following form:


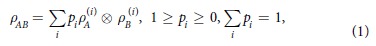


where *ρ*_*A*_^(*i*)^ and *ρ*_*B*_^(*i*)^ are local states of subsystems *A* and *B*, respectively.

The discussed entanglement of two quantum systems is a well understood concept, at least as far as the most simple case of two systems with two-dimensional state spaces (qubits) is concerned. However, the situation changes dramatically for three qubits. This is because three qubits can be grouped into two groups in three different ways and therefore we can divide their states into five classes according to their separability properties with respect to the particular bipartite splittings^3^. Thus apart from fully separable states which can be prepared by LOCC and fully inseparable states which are entangled across all three splittings, also partially entangled states exist which have some splittings separable. Although new phenomena^4^,^5^,^6^ and protocols^7^ can be found also for partially entangled states, most of the applications utilize fully inseparable states. This involves, for example, teleportation-based construction of quantum gates^8^ and protocol for quantum secret sharing^9^ as well as assisted teleportation^10^ or assisted dense coding^11^.

All previous applications rely on fully inseparable three-qubit Greenberger-Horne-Zeilinger (GHZ) state^12^


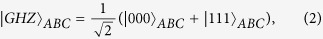


where the first, second and third qubit has been denoted as *A, B* and *C*, respectively. Except for gate construction^8^ all the applications are based on a genuine multipartite property of the GHZ state called localizability of entanglement^13^,^14^. Let us imagine, that three parties called Alice, Bob and Clare hold qubits *A, B* and *C* of GHZ state (2), respectively. Now, if Clare performs a suitable measurement on her qubit *C*, which does not reveal her any information about which of the two alternatives in the superposition on the right-hand side (RHS) of Eq. (2) took place, a maximally entangled state is localized between qubits *A* and *B*. More precisely, if Clare measures qubit *C* in basis |±〉_*C*_ = (|0〉_*C*_ ± |1〉_*C*_)/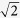 and finds an outcome “+”, a maximally entangled Bell state |Φ_+_〉_*AB*_ = (|00〉_*AB*_ + |11〉_*AB*_)/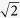 is established between Alice and Bob, whereas for outcome “−” the participants share another Bell state |Φ_−_〉_*AB*_ = (|00〉_*AB*_ − |11〉_*AB*_)/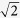. If Clare communicates the measurement outcome to Bob and he applies a phase flip on his qubit *B* if the outcome was “−”, Alice and Bob will share a single entangled state |Φ_+_〉_*AB*_, which they can subsequently use, e.g., for quantum teleportation^15^ or dense coding^16^. Localization of a two-qubit entanglement by a measurement of a qubit from a three-qubit GHZ state has been realized experimentally with trapped ions^17^, whereas assisted teleportation has been implemented with polarization-entangled photons^18^.

While for GHZ state (2) the amount of localized entanglement is maximal for all measurement outcomes there are other three-qubit pure states for which this is not the case anymore. The localizability of entanglement of these states can be characterized by an entanglement quantifier called entanglement of assistance^19^ defined as an average entropy of entanglement that can be localized between qubits *A* and *B* by a projective measurement on qubit *C*, which is maximized over all the measurements. In practice, a better established quantity called localizable entanglement is commonly used being a multipartite^13^ and mixed-state^14^ generalization of the entanglement of assistance, to which we will therefore refer also in what follows.

We have seen that localizability of entanglement is a key property for performance of several assisted quantum information protocols and it stays behind introduction of some entanglement quantifiers ranging from entanglement of assistance^19^,^20^ and localizable entanglement^13^,^14^ to entanglement of collaboration^21^. Besides that, localizability of maximum entanglement between any two qubits is an essential feature of cluster states being a backbone of the measurement-based model of quantum computation^22^,^23^. What is more, the possibility to localize maximal entanglement between at least one pair of qubits in a multiqubit pure state is a necessary condition for the state to be a universal resource for this model of quantum computation^24^. From the point of view of utility of a generic multipartite entangled state in previous applications it is important to know, whether the state contains localizable entanglement. Although a complete characterization of the set of states with localizable entanglement is a daunting task even for three-qubit states, it might still be possible to draw some conclusions about its structure. Clearly, the first logical step is to elucidate a more simple question as to whether a non-empty complement of the set of three-qubit states with localizable entanglement exists, i.e., whether there are some three-qubit entangled states for which two-qubit entanglement cannot be localized by any measurement on the remaining qubit.

Apparently, one can find some trivial examples of tripartite entangled states with nonlocalizable entanglement. For instance, if a tripartite entangled state is separable across some bipartite splitting, then its entanglement cannot be localized by any measurement on a part belonging to the bipartite part of the splitting. Similarly, if the latter state is separable with respect to at least two bipartite splittings its entanglement cannot be localized by any measurement on any of its parts. In all these cases, nonlocalizability of entanglement occurs in partially entangled states and it is a direct consequence of the separability properties of the respective states. Recently, tripartite entangled states with one and two separable bipartite splittings have been demonstrated experimentally both for qubits^25^ as well as for Gaussian states^26^,^27^,^28^.

In this paper we investigate both theoretically and experimentally the little explored area of states with *nonlocalizable* entanglement. Unlike above-mentioned partially entangled states with nonlocalizable entanglement, here we are interested in existence of nonlocalizable entanglement in the from the point of view of applications most important class of fully inseparable three-qubit states. Out of these states we primarily focus on states for which entanglement cannot be localized between *any* pair of qubits by any measurement on the third qubit. Instead of analyzing states with zero localizable entanglement, we analyze a subset of this set of states given by states for which entanglement cannot be localized probabilistically by any measurement (see Fig. 1). Very recently, an example of such a state has been constructed^29^ in the context of determination of genuine multipartite entanglement only from two-qubit reductions of the state. The state of ref. 29 is a genuine three-qubit entangled state with nonlocalizable entanglement, for which entanglement can be certified only from its two-qubit reductions. Since the state embodies two properties, it is rather complex and a natural question arises, whether nonlocalizable entanglement itself can be demonstrated with less complicated fully inseparable states. Here, we show that this is really the case. We prove, that nonlocalizable entanglement exists already in suitable three-qubit convex mixtures of GHZ state (2) and a maximally mixed state. We further generalize the latter set of states with nonlocalizable entanglement to a two-parametric family of states by adding a classically correlated fully separable state to the GHZ state. Next, we show that one can localize both conditionally as well as unconditionally the nonlocalizable entanglement with the help of the collective controlled-not (CNOT) operation. Moreover, we also prepare experimentally a state from the family using single photons and linear optical circuit and we prove that its entanglement cannot be localized by any measurement on any of the qubits. Finally, we conclude our experimental analysis of nonlocalizable entanglement by extracting a two-qubit entanglement from the experimentally prepared state using CNOT operation and postselection.

## Results

### Fully inseparable three-qubit state with nonlocalizable entanglement

The presence of nonlocalizable entanglement in a fully inseparable three-qubit state can be demonstrated on a very simple example of a convex mixture of the GHZ state (2) and the maximally mixed state (1/8)
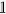

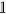
,


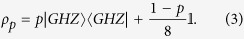


Here, the parameter *p* ∈ [0, 1] controls the ratio between the two states, the symbol 𝟙 denotes the 8 × 8 identity matrix, and we have suppressed the qubit indexes for brevity. Since the state is symmetric under the exchange of any two qubits, it is sufficient to investigate the presence of entanglement and its localizability only with respect to one bipartite splitting, say *C* − (*AB*) splitting. For certification of entanglement we can use the partial transposition criterion^30^,^31^ according to which in order qubit *C* to be entangled with a pair of qubits (*AB*) it is sufficient if the partial transpose of density matrix (3) with respect to the qubit (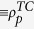) has a negative eigenvalue. Because state (3) belongs to the class of three-qubit generalizations of the Werner state^2^,^3^, the condition is also necessary and it reveals that the state is entangled if and only if *p* > 1/5^3^.

Moving to the analysis of the localizability of entanglement carried by state (3) let us assume that the last qubit *C* is projected onto a pure single-qubit state


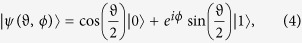


where *ϑ* ∈ [0, *π*] and *ϕ*∈[0, 2*π*). By calculating the (unnormalized) conditional state of qubits *A* and *B*, 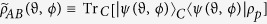, and normalizing it properly one finds, that the conditional state reads explicitly as


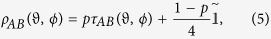


where


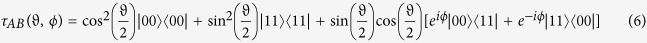


and 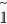 is the 4 × 4 identity matrix. Provided that there is *p* satisfying 1 ≥ *p* > 1/5 such that the conditional state (5) contains no entanglement for any *ϑ* and *ϕ*, the state (3) is a sought example of a fully inseparable state with nonlocalizable entanglement. To show that such the *p* really exists we use the two-qubit separability criterion which says that a density matrix *ρ*_*AB*_ is separable if and only if *det*(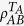) ≥ 0^32^. Applying the criterion to state (5) one finds after some algebra that


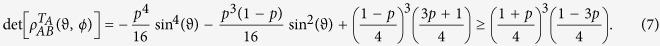


Hence we see, that if *p *≤ 1/3 the determinant is non-negative and thus the conditional state (5) is separable for any *ϑ* and *ϕ*. On the other hand, the lower bound on the RHS of inequality (7) is saturated for *ϑ* = *π*/2 and therefore in this case the determinant is negative for any *p* > 1/3. Thus we have arrived to the finding that for state (3) it is impossible to probabilistically localize entanglement between any pair of qubits by any projective measurement on the third qubit if and only if *p* ≤ 1/3. Since nonlocalizability of entanglement by any projective measurement implies its nonlocalizability by any generalized measurement (or even operation)^29^ we can conclude, that states (3), where parameter *p* lies in the interval 1/5 < *p* ≤ 1/3 are fully inseparable and their entanglement cannot be localized by any measurement.

A larger set of three-qubit fully inseparable states with nonlocalizable entanglement is obtained if the GHZ state on the RHS of Eq. (3) is replaced with the following convex mixture of GHZ state (2) and a classically correlated separable state,


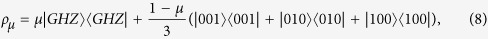


where *μ* ∈ [0, 1]. This gives the following two-parametric family of mixed three-qubit states


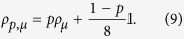


The state is again invariant under the exchange of any two qubits and thus also in this case it is sufficient to investigate whether the state is entangled and whether the entanglement is localizable only for *C* − (*AB*) splitting. Except for the extreme case when *μ* = 1 state (9) is not a three-qubit generalization of the Werner state^3^ anymore and the negativity of the partial transpose 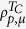 is known to be only sufficient for the presence of entanglement with respect to the splitting. Making use of the criterion one finds after some algebra that partial transposition 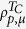 of state (9) possesses seven nonnegative eigenvalues and one eigenvalue equal to


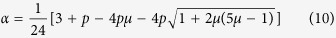


which can be nonnegative or negative. The state (9) contains entanglement across *C* − (*AB*) splitting and therefore it is fully inseparable due to the symmetry, if *α* < 0 which is equivalent with the following inequality:


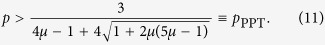


Localizability of entanglement carried by states (9) can be investigated analogously as in the case of states (3). After projection of last qubit *C* onto vector (4) density matrix (9) collapses into the state


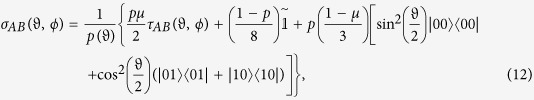


where state *τ*_*AB*_(*ϑ, ϕ*) is given in Eq. (6) and


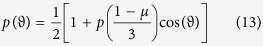


is a probability of detecting the state (4). In order to identify the region of parameters *p* and *μ* for which state (12) possesses no entanglement for any *ϑ* and *ϕ*, we again use the partial transposition criterion. Instead of investigating the sign of eigenvalues of matrix 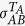 *(ϑ,ϕ*), it is more convenient to investigate the sign of eigenvalues of matrix 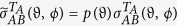, which possess the same sign owing to the inequality *p(ϑ*) > 0. The matrix 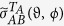 has three nonnegative eigenvalues and one eigenvalue


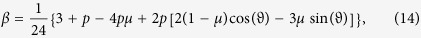


which can be nonnegative or negative. By solving extremal equation *dβ*/*dϑ* = 0 one finds that in the interior of the interval *ϑ* ∈ [0, *π*] eigenvalue (14) has one stationary point (≡*ϑ*_o*pt*_) which satisfies equation


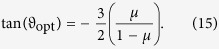


Hence, we get


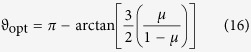


which gives


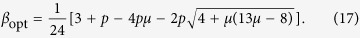


Comparison of the extremal eigenvalue with values of the eigenvalue (14) at the boundary points *ϑ* = 0 and *ϑ* = *π* of the interval [0, *π*] reveals, that it is not higher than the boundary values, and it is thus a global minimum on the interval. From condition *β*_o*pt*_ ≥ 0 we find, that the conditional state (12) is separable for any *ϑ* and *ϕ* if and only if


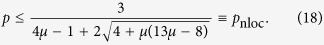


**Figure 2:**
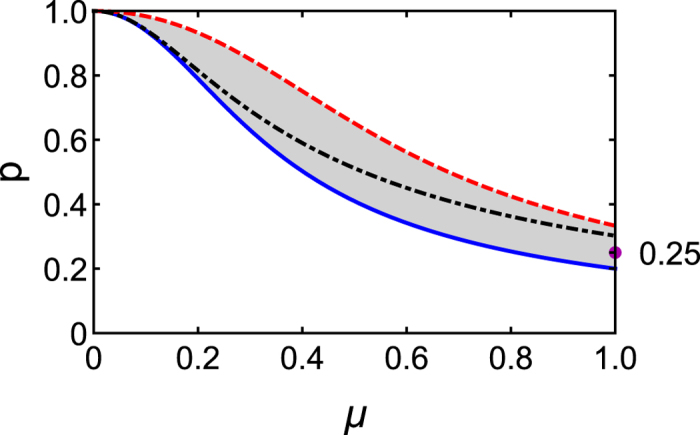
Dependence of *p*_PPT_, Eq. (11), (solid blue line), *p*_nloc_, Eq. (18), (dashed red line) and *p*_col_, Eq. (26), (dashed-dotted black line), as a function of parameter *μ*. Gray region depicts fully inseparable states (9) with nonlocalizable entanglement. The entanglement of all states above solid blue line can be transformed into two-qubit entanglement by the CNOT operation followed by conditioning on outcome “0” of the measurement in computational basis of output target qubit. The entanglement of all states above dashed-dotted black line can be transformed into two-qubit entanglement by the CNOT operation followed by trace-preserving operation (22). A dark magenta point on the right vertical axis depicts a state with nonlocalizable entanglement *ρ*_1/4,1_, Eq. (9), which we prepared experimentally. See text for details.

Consequently, state (9) for which parameter *p* satisfies inequalities *p*_P*PT*_ < *p* ≤ *p*_n*loc*_ is fully inseparable and its entanglement cannot be localized by any measurement. The region in the (*μ, p*)-plane of states (9) with the nonlocalizable entanglement is depicted by a gray color in Fig. 2.

### Entanglement localization by a collective operation

As we have already mentioned, the impossibility to localize entanglement of a tripartite quantum state by any projective measurement implies, that one cannot do that neither by any generalized measurement nor even by any probabilistic local operation^29^. This means, that in order to transform nonlocalizable entanglement in states (9) into two-qubit entanglement, local action on one qubit and classical communication to the locations of the other two qubits do not suffice, and some collective operation on several qubits is needed. In ref. 7 it was shown, that a two-qubit entanglement can be localized from a three-qubit partially entangled state by the CNOT operation followed by a suitable measurement on one output qubit or by a suitable trace-preserving operation on both output qubits. Inspired by this approach we show in the following subsection, that for all states with nonlocalizable entanglement investigated in previous section one can conditionally localize entanglement between qubits *A* and *B* by first letting qubit *C* to interact via the CNOT operation with qubit *B*, measuring qubit *C* in computational basis, and postselecting on projection onto state |0〉. The next subsection then deals with unconditional localization of entanglement between qubits *A* and *B*, which is reached by replacing the measurement with a suitable trace-preserving operation on qubits *B* and *C*.

### Conditional localization

Let us consider the CNOT operation described by the following unitary transformation,


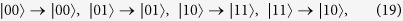


where the first and second qubit is called control and target qubit, respectively. Assume, that qubits *B* and *C* of state (9) interact via the CNOT operation, where qubit *B* is a control qubit and qubit *C* is a target qubit. The operation then transforms the state to


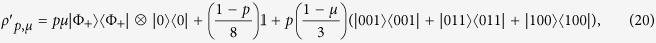


where |Φ_+_〉 is the maximally entangled Bell state defined below Eq. (2). If we further measure the last qubit *C* in the computational basis and we find it in state |0〉, the obtained (normalized) post-measurement state of qubits *A* and *B* attains the form


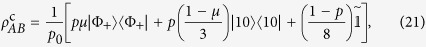


where *p*_0_ = [*p*(4*μ* − 1) + 3]/6 is the probability of finding state |0〉. Like previously we discuss separability properties of state (20) by applying the partial transposition criterion to unnormalized state 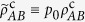. The partial transpose of the latter state with respect to the first qubit possesses three nonnegative eigenvalues and one eigenvalue (10) which is obviously negative if and only if *p* > *p*_PPT_, where *p*_PPT_ is defined in inequality (11). Hence we see, that for all entangled states (9) (including states with nonlocalizable entanglement), i.e., for all states lying above solid blue line in Fig. 2, it is indeed possible to localize entanglement by a CNOT operation on two qubits and postselection on a suitable outcome of a measurement in computation basis of one of the output qubits.

### Unconditional localization

There is yet another method of how one can extract nonlocalizable entanglement of states (9) between two qubits. In contrast with previous method it is unconditional and it relies on replacement of the measurement on qubit *C* behind the CNOT operation with a suitable trace-preserving completely positive map on qubits *B* and *C*. The map is described by the following Kraus operators^7^:


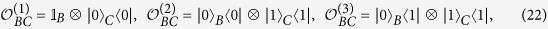


which satisfy the trace-preservation condition 
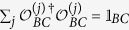
, where 𝟙_*B*_ (𝟙_*BC*_) is the single-qubit (two-qubit) identity matrix on the state space of qubit *B* (qubits *B* and *C*). If we transform by the map qubits *B* and *C* of the state after the CNOT operation, Eq. (20), we get


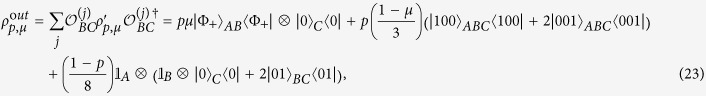


where 𝟙_*A*_ is the single-qubit identity matrix on the state space of qubit 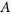. Further, by dropping qubit 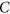 the remaining qubits *A* and *B* are left in the following state


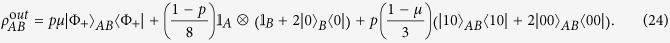


There are three nonnegative eigenvalues of the partial transpose 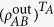 and one eigenvalue


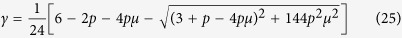


which is negative if and only if the parameter *p* satisfies inequality


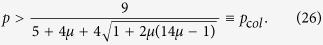


The lower bound 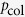 is depicted by the black dashed-dotted line in Fig. 2. The figure unveils that nonlocalizable entanglement of all states lying above the curve can be unconditionally transformed into two-qubit entanglement via the discussed collective operation on a pair of their qubits.

### Experiment

**Figure 3:**
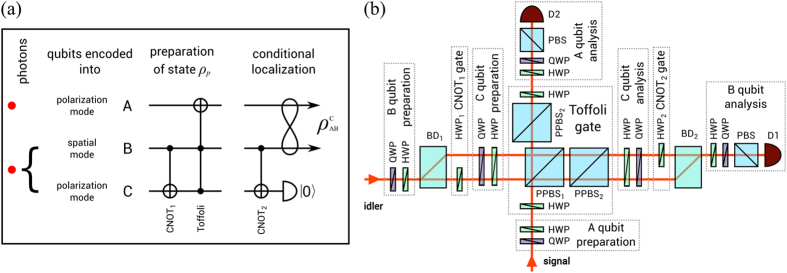
Experimental realization of a three-qubit fully inseparable state with nonlocalizable entanglement. Panel (a) shows encoding of three qubits *A,B* and *C* into two photons by exploiting their polarization and spatial degrees of freedom, a circuit for preparation of a state *ρ*_1/4,1_, Eq. (9), with nonlocalizable entanglement consisting of a two-qubit CNOT_1_ gate followed by a three-qubit Toffoli gate and a circuit for conditional localization of entanglement between qubits *A* and *B* by a CNOT_2_ gate on qubits *B* and *C* followed by a measurement on qubit *C*. Panel (b) shows a scheme of the experimental setup implementing state preparation and conditional entanglement localization from panel (a). The components are labelled as follows: QWP - quarter-wave plate, HWP - half-wave plate, BD - calcite beam displacer, PPBS - partially polarizing beam splitter, PBS - polarizing beam splitter, D - single-photon avalanche diode. See main text for more details.

We have also performed a proof-of-principle experiment demonstrating the existence of a three-qubit fully inseparable state with nonlocalizable entanglement. We have prepared and analyzed the state 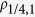, Eq. (9), using the circuits in Fig. 3 (a) implemented by linear optical setup shown in Fig. 3 (b). We have used orthogonally polarized time-correlated photon pairs generated in the process of spontaneous parametric down-conversion in a nonlinear crystal pumped by a cw laser diode^33^. The qubit 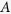 is encoded into the polarization degree of freedom of the signal photon whereas qubits *B* and *C* are encoded into the spatial and polarization degree of freedom, respectively, of the idler photon (see Fig. 3 (a)).

Polarization qubits *A* and *C* are prepared and analyzed by polarization measurement blocks consisting of a quarter-wave plate (QWP), half-wave plate (HWP), and a polarizing beam splitter (PBS) where computational states 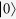 and 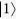 are represented by horizontal and vertical polarization, respectively. The qubit 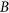 is initially prepared in polarization encoding using combination of QWP and HWP and it is subsequently converted into path encoding using a polarizing beam displacer (BD_1_). The computational state 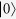 corresponds to the horizontally polarized photon propagating in the upper interferometer arm, while the state 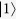 is represented by a vertically polarized photon propagating in the lower interferometer arm of an inherently stable Mach-Zehnder interferometer formed by two calcite beam displacers BD_1_ and BD_2_. Polarization to path conversion produces path-polarization entangled states which can be disentangled by HWP_1_ that addresses a single arm of the interferometer. The action of HWP_1_ can be regarded as a quantum CNOT_1_ gate acting on the spatial control and polarization target qubits. Beam displacer BD_2_ together with HWP_2_ map the spatial qubit back onto polarization one. The core of the setup is three-qubit Toffoli gate implemented by two-photon interference on a partially polarizing beam splitter (PPBS_1_) followed by two additional PPBSs which serve as partial polarization filters^34^,^35^,^36^,^37^. Please note, that Toffoli gate is equivalent to the three-qubit controlled-controlled-Z gate up to single-qubit Hadamard transforms on the target qubit (in our case qubit 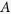). Our scheme is probabilistic and operates in coincidence basis where successful operation is heralded by detection of two-photon coincidences D1&D2 at the output. More details about experimental setup can be found in ref. 38.

To have full control over the structure of prepared states we have separately prepared GHZ state (2) and all states 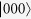, 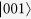, …, 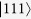 of the computational basis representing diagonal elements of the identity matrix. To generate GHZ state we have prepared qubit 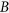 initially in |+〉 state and qubit 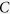 in |0〉 state. With suitable rotation of HWP_1_ we have created Bell state 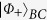 which interacts in Toffoli gate with qubit 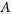 prepared in |0〉 state. The diagonal elements of the identity matrix have been prepared in a similar way.

**Figure 4:**
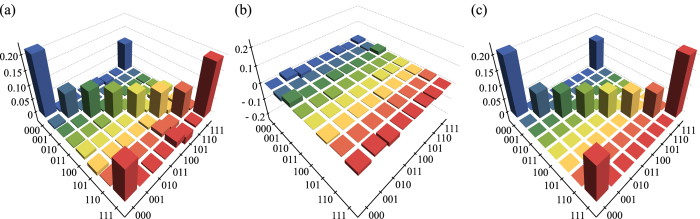
Real (a) and imaginary (b) part of the reconstructed density matrix *ρ*_exp_ and (c) density matrix of the ideal state *ρ*_1/4,1_. Note that the theoretical density matrix has only real values.

Each prepared state was characterized by three qubit quantum state tomography which consists of sequential projections onto the six states 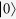, 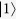, |+〉, |−〉, (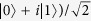, (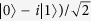 at each output qubit for total 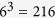 measurements. For each measurement two-photon coincidences were recorded for 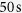. The measured coincidence counts were normalized by sum of all coincidences and relative frequencies were obtained. The state characterization lasted less than 4 hours. In order to demonstrate nonlocalizability of entanglement on a most simple state, we prepared a three-qubit state 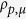, Eq. (9), with *μ* = 1, and to have a sufficiently robust effect against experimental imperfections, we have chosen 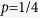, which guarantees that the state lies sufficiently deep inside the set of states with nonlocalizable entanglement (see dark magenta point in Fig. 2). The relative frequencies of the required state 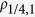 have been obtained by mixing relative frequencies of the GHZ state and diagonal elements of the identity matrix, and the state was reconstructed using the maximum likelihood estimation algorithm^39^,^40^. The reconstructed density matrix 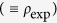 exhibits a large overlap with the ideal state 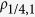 as is witnessed by a fidelity of 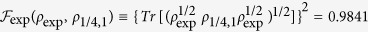. For estimation of statistical uncertainty of the experimental results we have used a standard Monte Carlo analysis. Using measured coincidence counts as a mean value of Poisson distribution we have numerically generated 1000 samples of the state 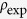, which were again reconstructed with the help of the maximum likelihood estimation algorithm. For the generated set of density matrices we obtained the average fidelity of 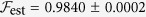, which is in an excellent agreement with the experimental value. To get a deeper insight into the structure of the experimentally prepared state 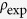 and to see its resemblance to the ideal state 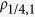 we display both states in Fig. 4.

In the next step of our analysis we certified full inseparability of the prepared state by calculating lowest eigenvalues 
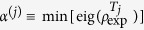
, 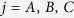, for all three partial transpositions of the experimental density matrix 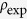. The errors have been again calculated with the help of the Monte Carlo method. Within the statistical uncertainty the experimental eigenvalues coincide with average eigenvalues obtained from the computationally generated population of the experimental density matrix. The eigenvalues together with the errors are summarized in Table 1.

**Figure 5:**
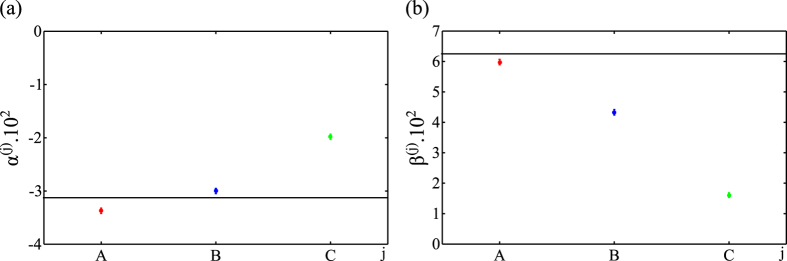
(a) Displays eigenvalues *α*^(*A*)^ (red), *α*^(*B*)^ (blue) and *α*^(*C*)^ (green) from Table 1. Solid line in (a) depicts the theoretical value of the eigenvalues, 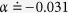. (b) Displays eigenvalues *β*^(*A*)^ (red), *β*^(*B*)^ (blue) and *β*^(*C*)^ (green) from Table 2. Solid line in (b) represents the theoretical value of the eigenvalues, 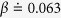. Error bars have been calculated by the Monte Carlo method. See text for details.

For a better illustration, we display the eigenvalues from Table 1 in Fig. 5 (a). Inspection of the figure unambiguously proves, that all the eigenvalues are many standard deviations below zero and therefore the prepared state is fully inseparable by the partial transposition criterion. Note further, that while according to the theory the eigenvalues should all be equal to a single value 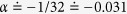, Eq. (10) with *μ* = 1 and *p* = 1/4, they increase as we go from qubit 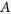 to qubit 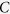 (see Fig. 5 (a)). This behaviour can be attributed to the fact that each qubit passes through different number of optical elements and thus suffers by different state-dependent losses. Indeed, a simple theoretical model of our operation consisting of an ideal Toffoli gate followed by local filters on each qubit qualitatively captures the behaviour of experimental eigenvalues and thus confirms this intuitive explanation.

In the final step, we have verified nonlocalizability of entanglement in the prepared state again using partial transposition criterion. First, we have calculated from the experimental density matrix *ρ*_exp_ the (normalized) conditional state (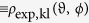) of qubits *k* and *l, k* ≠ *l* = *A, B, C*, after projection of qubit *j* ≠ *k, l* of the state *ρ*_exp_ onto pure state (4). Next, we have calculated the optimized eigenvalue 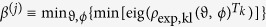, *j* = *A, B*, C, by numerical minimization of the lowest eigenvalue of the partial transpose of the conditional state 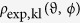 with respect to qubit *k* over parameters *ϑ* and *ϕ*. Further, making use once again the Monte Carlo analysis we have also calculated averages of the eigenvalues *β*^(*j*)^ and the corresponding errors. Like in the previous case, the experimental eigenvalues were found to be equal to the average eigenvalues within the presented accuracy. The experimental eigenvalues with errors are summarized in Table 2.

The eigenvalues from Table 2 are displayed in Fig. 5 (b). The figure reveals that all the eigenvalues lie by many standard deviations above zero and thus the entanglement carried by the experimentally prepared state is indeed nonlocalizable by any measurement. Similar to eigenvalues certifying full inseparability, also the experimental eigenvalues from Fig. 5 (b) differ from the theoretical eigenvalue 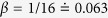. This behaviour can be again attributed to the previously discussed imperfect realization of Toffoli gate.

We have accomplished experimental investigation of the concept of nonlocalizable entanglement by extracting conditionally two-qubit entanglement from the prepared state *ρ*_1/4,1_ via CNOT_2_ operation and postselection as described in subsection dedicated to localization of entanglement by a collective operation. The preparation of state *ρ*_1/4,1_ was the same as described above. Next, we have performed CNOT_2_ operation using HWP_2_ on qubits *B* and *C*, where qubit *B* is a control qubit and qubit *C* a target qubit. Finally, we have performed projection of qubit *C* onto the |0〉 basis state. Acquired data were processed as in the case of preparation of state *ρ*_1/4,1_. The obtained conditional two-qubit state 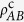, Eq. (21) with *p* = 1/4 and *μ* = 1, has been again characterized by the quantum state tomography. The lowest eigenvalue of the partial transpose 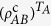 of the reconstructed density matrix 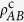 reads 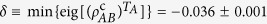. The negativity of the eigenvalue clearly confirms successful conditional localization of two-qubit entanglement by a collective operation on a pair of qubits of the prepared state *ρ*_1/4,1_. Note finally, that owing to experimental imperfections the experimental eigenvalue *δ* is larger than the ideal theoretical value of the eigenvalue of *α*/*p*_*0*_ = −1/20 = −0.05, where we used Eq. (10) and the formula for success probability *p*_0_ below Eq. (21) with *μ* = 1 and *p* = 1/4.

## Discussion

We have proposed and experimentally demonstrated the concept of nonlocalizable entanglement in the context of three-qubit fully inseparable states. This type of entanglement is confined in a three-qubit system such, that no measurement on either of the qubits is capable to localize entanglement between the remaining two qubits. Because this property guarantees that also no probabilistic operation can accomplish this^29^, there is no way of how one could turn three-qubit entanglement of the state into a useful two-qubit entanglement by using local operation on one qubit and postselecting the other two qubits on successful realization of the operation. In this respect, nonlocalizable entanglement resembles nondistillable (bound) entanglement carried by tripartite states for which three parties holding parts of such a state cannot establish entanglement between any two of them with the help of the third one by LOCC^3^,^4^. In fact, for qubit states localizability of entanglement suffices for distillability. This is because by measuring suitably one qubit of a three-qubit state with localizable entanglement we create entanglement between the remaining two qubits which is always distillable^41^. Our results further show, that localizability of entanglement is not necessary for distillability as there exist states with nonlocalizable entanglement which are distillable. As an example of such a state can serve us mixtures *ρ*_*p*_, Eq. (3), of the GHZ state and a maximally mixed state with 1/5 < *p* ≤ 1/3, from which we have prepared the state wit *p* = 1/4 experimentally. According to our results, such states carry nonlocalizable entanglement because they do not surpass the nonlocalizability bound *p*_nloc_ = 1/3 and at the same time they are distillable, as all the states with *p* > 1/5 are distillable^3^. The existence of such states is expectable, because we consider localization of entanglement by local operations on one part of a single copy of the state followed by a postselection of the other two parts, whereas for entanglement distillation more powerful LOCC operations acting on all three parts of generally multiple copies of the state are used. The question of the existence of states with single-copy nonlocalizable entanglement which would be localizable when two or more copies are measured jointly is deferred for further research. Their existence is likely because one can find three-qubit states which give strictly more than two times larger average entropy of entanglement that can be localized by a measurement on two copies of the state than that of one can localize on a single copy^19^.

We have seen that extraction of two-qubit entanglement from three-qubit states with nonlocalizable entanglement would require more copies of the state and more powerful LOCC operations. Another option is to work only with a single copy of the state but resign on the LOCC character of the used method. Here, we have proposed for the investigated family of states both conditional and unconditional localization method based on application of the CNOT operation on a pair of qubits of the state followed by a measurement on one output of the operation and trace-preserving operation on both outputs, respectively. Additionally, for the prepared mixture we have also realized the more simple conditional method experimentally. Needles to say finally, that the investigated mixtures *ρ*_*p*_ with nonlocalizable entanglement do not possess the strongest form of multipartite entanglement which usually appears in applications. The true is that the states are entangled with respect to all three bipartite splittings and thus they are fully inseparable. On the other hand, the states satisfy a necessary and sufficient condition for biseparability *p* ≤ 3/7^42^, and therefore they can be created by mixing of three-qubit entangled states which are separable across different bipartite splittings. The strongest form of multipartite entanglement, the so called genuine multipartite entanglement, is carried by states which are not biseparable. To demonstrate the existence of nonlocalizable genuine multipartite entanglement we would have to leave the set of states *ρ*_*p*_ in which the two properties never coexist. To the best of our knowledge, there is currently known one example of a state which carries simultaneously both nonlocalizable and genuine multipartite entanglement^29^. However, an experimental demonstration of such a state would be much more challenging in comparison with the states investigated here owing to the need to prepare large coherent superpositions of three-qubit computational basis states with precisely adjusted absolute values and phases of nontrivial complex amplitudes. We hope that our findings will stimulate further investigation of multipartite fully inseparable states which are too noisy to possess localizable entanglement but which are not noisy enough to be nondistillable.

## Additional Information

**How to cite this article:** Mičuda, M. *et al*. Experimental demonstration of a fully inseparable quantum state with nonlocalizable entanglement. *Sci. Rep.* **7**, 45045; doi: 10.1038/srep45045 (2017).

**Publisher's note:** Springer Nature remains neutral with regard to jurisdictional claims in published maps and institutional affiliations.

## Figures and Tables

**Figure 1:**
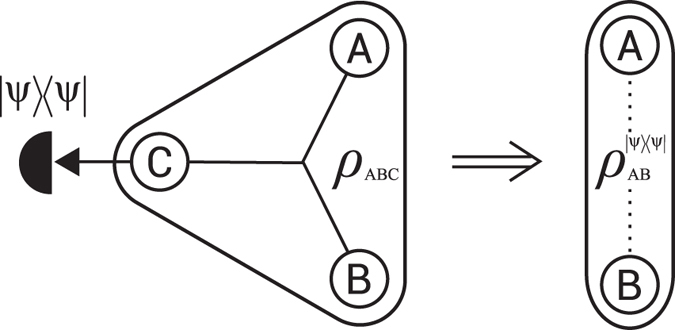
A scheme depicting nonlocalizability of entanglement in a fully inseparable state *ρ*_*ABC*_ of three qubits *A, B* and *C*. For any projection of qubit *C* onto a pure state |*ψ*〉 the resulting two-qubit state 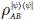 is separable. See text for details.

**Table 1:** Minimal eigenvalue *α*^(*j*)^ with one standard deviation of the partial transpose with respect to qubit *j* of the experimental density matrix *ρ*_exp_.

*j*	A	B	C
*α*^(*j*)^.10^2^	−3.37 ± 0.05	−3.00 ± 0.05	−1.98 ± 0.05

Errors have been obtained using the Monte Carlo method.

**Table 2:** Optimized eigenvalue *β*^(*j*)^ with one standard deviation, which is given by the minimum over all *ϑ*∈[0, π] and *ϕ* ∈ (0, 2π) of the lowest eigenvalue of the partial transpose with respect to first qubit of a conditional state obtained by projection of qubit *j* of the experimental density matrix *ρ*_*exp*_ onto pure state (4).

*j*	A	B	C
*β*^(*j*)^.10^2^	5.98 ± 0.09	4.33 ± 0.09	1.61 ± 0.08

Errors have been calculated using the Monte Carlo method.
